# Comparison of total intravenous anesthesia and combined inhalation and intravenous anesthesia on survival after tumor surgery: a propensity score matched cohort study

**DOI:** 10.3389/fmed.2026.1659919

**Published:** 2026-02-11

**Authors:** Yu Wang, Xi Wu, Shuaijie Pei, Yangqi Chu, Yiyi Yang, Yun Lin, Demu Ba, Peng Yin, Jie Wang, Shanglong Yao

**Affiliations:** 1 Department of Anesthesiology, Union Hospital, Tongji Medical College, Huazhong University of Science and Technology, Wuhan, China; 2 Institute of Anesthesia and Critical Care Medicine, Union Hospital, Tongji Medical College, Huazhong University of Science and Technology, Wuhan, China; 3 Key Laboratory of Anesthesiology and Resuscitation (Huazhong University of Science and Technology), Ministry of Education, Wuhan, China; 4 Department of Critical Care Medicine, Tianjin Medical University General Hospital, Tianjin, China; 5 Department of Anesthesiology, Tianjin Medical University General Hospital, Tianjin, China; 6 Department of Anesthesiology, People’s Hospital of Bozhou, Bole, Xinjiang, China; 7 National Center for Chronic and Noncommunicable Disease Control and Prevention, Chinese Center for Disease Control and Prevention, Beijing, China; 8 Hubei Clinical Research Center of Anesthesiology, Hubei, China

**Keywords:** combined inhalation and intravenous anesthesia, propensity score matching, short/long-term mortality, total intravenous anesthesia, tumor resection

## Abstract

**Background:**

Prior studies have indicated that inhalation anesthesia in cancer surgery might heighten the risk of tumor metastasis and diminish patient survival. Yet, combined inhalation and intravenous anesthesia (CIVA), a prevalent general anesthesia technique, remains underexplored regarding its influence on postoperative survival in cancer surgery patients. To fill this gap, this study compares the short-term and long-term mortality of cancer surgery patients receiving CIVA versus total intravenous anesthesia (TIVA).

**Methods:**

A retrospective cohort study was conducted at a tertiary care hospital in China, comprising 25,351 patients who underwent cancer surgery under general anesthesia between January 2014 and December 2018. The primary outcomes were short-term mortality (within 3 months) and long-term mortality (within 3 years). CIVA and TIVA were the primary exposures. Propensity score matching (PSM) was employed to adjust for confounding factors, and Cox regression models were utilized to estimate hazard ratios (HR) and 95% confidence intervals (CIs) for mortality outcomes.

**Results:**

Among the 25,351 patients who underwent tumor resection, 23,790 were administered TIVA, while 1,561 received CIVA. In the 1:3 PSM cohort, 1,536 patients received CIVA, and 4,519 patients received TIVA. The Cox regression models for the 1:1, 1:2, and 1:3 PSM cohorts indicated that CIVA was associated with long-term mortality but not with short-term mortality. The multivariable Cox regression model following 1:3 PSM revealed that CIVA was associated with an increased risk of 3-year mortality (HR: 1.220; 95% CI: 1.043–1.404).

**Conclusion:**

Our results provided indirect evidence of potential hazard of inhaled anesthetics, even as a compound in CIVA, on long-term mortality after cancer surgery. Given the limitations of this retrospective study, further prospective work exploring the effect of anesthetic technique on mortality is urgently needed.

## Introduction

Amid the backdrop of a growing elderly demographic, the prevalence of cancer is projected to escalate from 14 million cases in 2012 to approximately 24 million cases by 2035 (1). For patients with solid organ cancers, surgery serves as a pivotal treatment modality, with estimates suggesting that surgical procedures will be integral to the treatment plans of more than 80% of cancer patients (2). Paradoxically, surgery itself, surgical stress response, and anesthesia treatments can facilitate tumor proliferation or metastasis by multiple pathways, such as metabolic and neuroendocrine perturbations (3–7). This has led to increasing interest in the impact of anesthesia and operation on cancer progression and mortality (8).

Over the past decade, the impact of anesthesia on cancer recurrence has garnered growing interest, prompting a surge in retrospective studies and pre-clinical investigations. Various underlying biological mechanisms have been proposed to explain how different anesthetic agents might affect cancer outcomes involved in tumorigenesis and metastasis (9, 10). In clinical practice, there are two key classes of general anesthetic agents: intravenous propofol and inhalational volatile anesthetics. Inhalational volatile anesthetic agents like sevoflurane, potentially augment proliferation, migration, invasion, and angiogenesis in various cancer cell types (9), while propofol has been shown to counteract these pathways (10, 11). Given these properties, it is theoretically possible that inhalational volatile anesthetics could potentially promote tumor metastasis and recurrence.

Unfortunately, the available evidence regarding the effects of specific anesthetic agents on cancer outcomes, are inconclusive (7, 8, 12–15). Some researchers showed that volatile anesthesia could damage the cancer-specific immune defenses *in vitro* study (9), and increase the mortality after cancer surgery in retrospective clinical study (7), compared with intravenous anesthetic agents. Yet, others did not observe a significant association between the type of general anesthesia and overall mortality after cancer surgery (12, 13). The inconsistent findings regarding the impact of inhalation and intravenous anesthesia on postoperative cancer survival can be largely attributed to the fact that the majority of studies conducted thus far are retrospective cohort studies, which are prone to issues such as incomplete data, selection bias, and confounding factors. Moreover, the inherent biological characteristics and prognostic factors vary significantly among different types of cancer, such as breast cancer, colorectal cancer, and lung cancer, adding another layer of complexity to the research outcomes. Furthermore, we speculate that regional differences in the types, dosages, and durations of anesthetic agents used in clinical practice may also exert a certain influence on postoperative prognosis.

General anesthesia is presently offered in a variety of forms, including total intravenous anesthesia (TIVA), fluorinated inhalation anesthesia (IA) and combined inhalation and intravenous anesthesia (CIVA). Previous clinical studies have focused on the effects of TIVA and inhalation anesthesia on mortality. Among the published evidence, there are currently no data concerned the effect of CIVA or TIVA on mortality of cancer patient. To further assess the relationship between type of anesthesia and mortality after oncologic surgery, we conducted this retrospective cohort study to compare the short/long-term mortality after cancer surgery in patients receiving CIVA or TIVA.

## Methods

### Study participants

**Figure 1:**
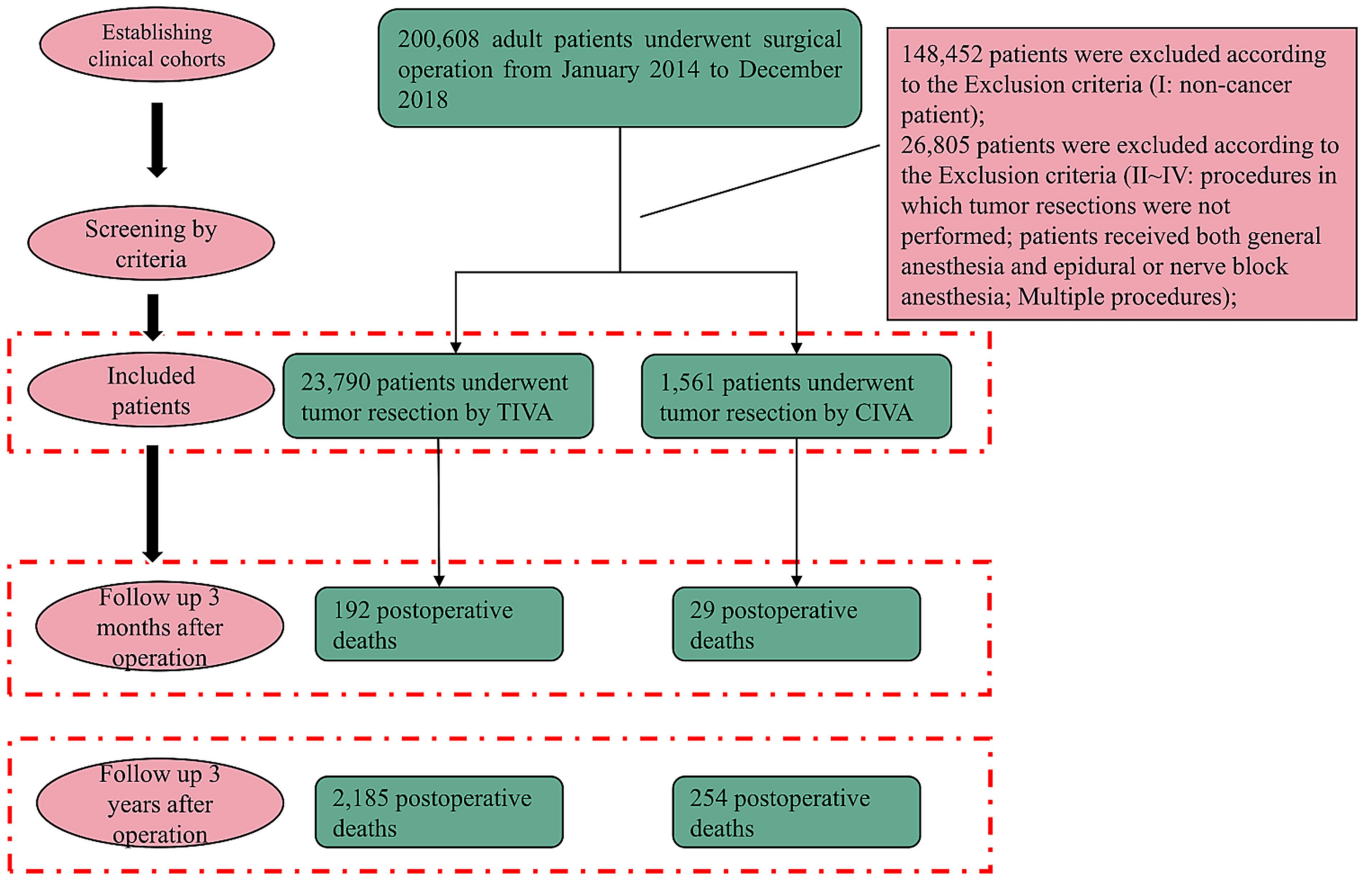
Flowchart of patient selection. Exclusions: (I) non-cancer patient; (II) endoscopic polyp resections, intestinal stent insertions, and procedures in which tumor resections were not performed; (III) combined anesthesia; (IV) multiple procedures with different anesthesia forms.

This retrospective cohort study was conducted at Wuhan Union hospital, Hubei, China, a tertiary comprehensive hospital, following the Strengthening the Reporting of Observational Studies in Epidemiology (STROBE) reporting guideline. This study was authorized by the ethical committee at Union Hospital, Tongji Medical College, Huazhong University of Science and Technology (20210641) on 19th July, 2023. The committee did not require written informed consent since only aggregated data that was not personally identifiable to the patient was used. All patients presenting for cancer surgery over a five-year period (January 2014 to December 2018) who required general anesthesia were included (Figure 1). Cancer diagnoses were confirmed based on postoperative pathological reports, ensuring accurate identification of tumor characteristics. The exclusion criteria are as follows: (I) Non-cancer patient; (II) Procedures in which tumor resections were not performed; (III) Patients received both general anesthesia and epidural or nerve block anesthesia; (IV) multiple procedures.

### Exposure

The two forms of general anesthesia of interest in this study were TIVA and CIVA. TIVA was identified as relying on sustained infusion of propofol and remifentanil to maintain general anesthesia. In contrast, CIVA technique was defined as the concurrent administration of any inhaled volatile anesthetic (sevoflurane or desflurane) with intravenous propofol and remifentanil for >15 min during the maintenance period. The exact concentrations and the proportional maintenance time contributed by inhalation vs. intravenous agents were not captured in our dataset.

### Outcomes

We extracted information from electronic clinical medical records of Wuhan Union Hospital. The primary outcome was cancer-cause mortality derived from the China’s National Disease Surveillance Points (DSP) system spanning the years 2014 to 2021. It was defined as death with cancer as the root cause, recorded in the Life Registration and Cause of Death Monitoring Room, China CDC, which excluded death directly caused by cardiogenic disease and trauma. According to the survival time of patients, the death outcome was further divided into short-term death (3 months after operation) and long-term death (3 years after operation).

This DSP system is established and managed by the Chinese Center for Disease Control and Prevention (China CDC). After a significant upgrade in 2013, the DSP system expanded its surveillance points to 605, covering urban (208 points) and rural (397 points) areas across all 31 provinces/autonomous regions/municipalities (hereafter termed provinces) of the Chinese mainland (16). Mortality data are collected from a wide range of sources, encompassing hospitals, private residences, and various other locations. Trained professionals based at local hospitals or branches of the CDC utilize a standardized protocol to ascertain the causes of death. The DSP system has been validated in prior research as a robust tool for capturing both national and regional data, reliably documenting cancer-related fatalities (17, 18). To maintain the integrity and consistency of the data, annual training sessions are implemented, focusing on the standardized protocol. These sessions incorporate random audits to verify the precision of disease classification and to identify any duplications. Additionally, retrospective reviews are periodically conducted to assess the extent of underreporting and to ensure the accuracy and completeness of disease coding. Every 3 years, underreporting surveys are executed to evaluate the DSP system’s comprehensiveness and to rectify any potential biases stemming from underreporting (19).

### Matching covariates

The confounding variables for propensity score matching (PSM) included in this study were sex, age, body mass index (BMI), American society of Anesthesiologists physical status (ASAPS), duration of surgery, type of tumor (including ear, nose & throat [ENT] tumor, Gastrointestinal tract tumor, Orthopedic tumor, Urological tumor, General surgery tumor, Nervous system tumor, Thoracic tumor, and Ophthalmic tumor), previous surgical history, previous heart disease history, previous diabetes history, previous hypertension history, previous cerebral infarction history, history of drinking, history of smoking, surgery grade, emergency surgery, shift change of anesthesiologist, transfusion, intraoperative blood loss, intraoperative blood transfusion volume and intraoperative vasoactive drugs. Detailed definitions of matching variables are provided in Supplementary Method 1.

### Statistical analysis

Clinical data are presented as numbers (percentage) for categorical variables and as mean ± standard deviation (SD) or median (minimum, maximum) for continuous variables, as appropriate. Preliminary assessments of differences in clinical data were conducted using Student’s t-test or Welch’s *t*-test for continuous variables, and Pearson’s chi-squared test or Fisher’s exact test for categorical variables, between the TIVA and CIVA groups before and after PSM.

The PSM was applied to balance confounding variables between the TIVA and CIVA groups in this study. A multivariable logistic regression model was used to estimate the propensity scores. The 1:1, 1:2, and 1:3 matching protocols without replacement, along with a caliper of 0.05 SD of the logit of the propensity score, were employed. Standardized mean differences (SMDs) were computed for the three PSM cohorts to assess baseline balance following PSM. Cox regression models were applied to the three PSM cohorts to calculate hazard ratios (HRs) and their associated 95% confidence intervals (CIs) for long- and short-term postoperative mortality. Univariate and multivariable Cox regression models were used to investigate the TIVA/CIVA associated with postoperative mortality. Confounding variables with *p* < 0.05, identified between the TIVA and CIVA groups after 1:3 PSM, were included in the multivariable Cox regression analyses to calculate the adjusted HR.

All statistical analyses were conducted using the R environment, version 4.2.0. The statistical packages “MatchIt” and “survival” were used for propensity score matching and Cox regression, respectively (Supplementary Method 2).

## Results

### Study population and baseline characteristics

A total of 200,608 patients who received surgical operation under general anesthesia in Wuhan Union Hospital between January 2014 and December 2018 were identified. 148,452 non-cancer patients were excluded. In the remaining population, 26,805 patients were removed based on exclusion criteria II–IV. Ultimately, 23,790 (93.8%) patients were included in the TIVA group while 1,561 (6.2%) patients were included in the CIVA group (Table 1). Data before PSM demonstrated that when compared to patients received TIVA, patients who received CIVA had a higher incidence of 3-year mortality (254 [16.3%] vs. 2,185 [9.2%]) and 3-month mortality after operation (29 [1.9%] vs. 192 [0.8%]).

**Table 1:** Data of cancer patients undergoing tumor resection before propensity score matching.

	Before propensity score matching				
	TIVA (*N* = 23,790)	CIVA (*N* = 1,561)	*p*-value	SMD	SMD-0.1
Outcomes					
Death 3 years after operation	2,185 (9.2%)	254 (16.3%)	<0.001	–	–
Death 3 months after operation	192 (0.8%)	29 (1.9%)	<0.001	–	–
Matching variables					
Age	49.0 (15.2)	51.0 (20.5)	<0.001	0.10	<0.1
Male	10,059 (42.3%)	824 (52.8%)	<0.001	0.21	>0.1
BMI	23.8 (4.41)	23.6 (5.22)	0.208	0.03	<0.1
ASAPS					
I	1,586 (6.7%)	129 (8.3%)	<0.001	0.06	<0.1
II	11,018 (46.3%)	1,022 (65.5%)		0.40	>0.1
III	3,570 (15.0%)	375 (24.0%)		0.21	>0.1
IV and higher	7,616 (32.0%)	35 (2.2%)		2.23	>0.1
Duration of surgery	2.40 [0, 23.5]	3.25 [0, 13.1]	<0.001	0.39	>0.1
Tumor type					
ENT tumor	1776 (7.5%)	19 (1.2%)	<0.001	0.57	>0.1
Gynecological tumor	1,216 (5.1%)	91 (5.8%)		0.03	<0.1
Gastrointestinal tract tumor	5,694 (23.9%)	627 (40.2%)		0.33	>0.1
Orthopedic tumor	1,646 (6.9%)	87 (5.6%)		0.06	<0.1
Urological tumor	1,616 (6.8%)	147 (9.4%)		0.09	<0.1
General surgery tumor	8,666 (36.4%)	354 (22.7%)		0.33	>0.1
Nervous system tumor	2,171 (9.1%)	141 (9.0%)		0.00	<0.1
Thoracic tumor	896 (3.8%)	78 (5.0%)		0.06	<0.1
Ophthalmic tumor	109 (0.5%)	17 (1.1%)		0.06	<0.1
Previous surgical history	8,756 (36.8%)	509 (32.6%)		0.09	<0.1
Previous heart disease	318 (1.3%)	66 (4.2%)	<0.001	0.14	>0.1
Previous diabetes	1,041 (4.4%)	114 (7.3%)	<0.001	0.11	>0.1
Previous hypertension	3,530 (14.8%)	353 (22.6%)	<0.001	0.19	>0.1
Previous cerebral infarction	198 (0.8%)	28 (1.8%)	<0.001	0.07	<0.1
History of drinking	2,624 (11.0%)	201 (12.9%)	<0.001	0.06	<0.1
History of smoking	3,584 (15.1%)	318 (20.4%)	0.027	0.13	>0.1
Surgery grade			<0.001		
Intermediate	10,172 (42.8%)	952 (61.0%)		0.37	>0.1
Major	3,067 (12.9%)	219 (14.0%)	<0.001	0.03	<0.1
Minor	10,551 (44.4%)	390 (25.0%)		0.45	>0.1
Emergency surgery	2,864 (12.0%)	188 (12.0%)		0.00	<0.1
Shift change of anesthesiologist	3,627 (15.2%)	341 (21.8%)	1	0.10	>0.1
Night shift (18:00 ~ 8:00)	922 (3.9%)	100 (6.4%)	<0.001	0.16	>0.1
Transfusion	1,229 (5.2%)	281 (18.0%)	<0.001	0.33	>0.1
Intraoperative blood loss	68.4 (267)	150 (439)	<0.001	0.19	>0.1
Intraoperative blood transfusion amount	30.6 (145)	75.3 (293)	<0.001	0.15	>0.1
Intraoperative vasoactive drugs	2,784 (11.7%)	395 (25.3%)	<0.001	0.31	>0.1

TIVA = total intravenous anesthesia; CIVA = combined inhalation and intravenous anesthesia; SMD=Standard mean deviation; BMI=Body Mass index; ASAPS = American society of Anesthesiologists physical status; ENT surgery = ear, nose, and throat surgery. Data are presented as the number (percentage) for categorical variables and as the mean ± SD or median (minimum, maximum) for continuous variables, as appropriate. The independent samples *t*-test was used to compare continuous variables and the chi-square test to compare categorical variables between groups.

Regarding baseline characteristics, patients in the CIVA group were older (mean [SD] age, 51.0 [20.5] years vs. 49.0 [15.2] years), more likely to be male (52.8% vs. 42.3%) and had a higher proportion of preoperative comorbidities (heart disease: 4.2% vs. 1.3%; diabetes: 7.3% vs. 4.4%; hypertension: 22.6% vs. 14.8%; cerebral infarction: 1.8% vs. 0.8%). In terms of anesthesia, patients with ASAPS I-III tended to receive CIVA (I: 8.3% vs. 6.7%; II: 65.5% vs. 46.3%; III: 24.0% vs. 15.0%), whereas a higher proportion of patients with ASAPS IV and above received TIVA (32.0% vs. 2.2%). Patients in the CIVA group had a higher percentage of vasoactive drug usage (25.3% vs. 11.7%) and transfusion (18.0% vs. 5.2%), as well as more intraoperative blood loss (mean [SD]: 150 [439] vs. 68.4 [267]) and transfusion amount (mean [SD]: 75.3 [293] vs. 30.6 [145]). In addition, patients in the CIVA group had longer operating times (median [minimum, maximum], 3.25 [0, 13.1] vs. 2.40 [0, 23.5]) and were more likely to undergo intermediate (61.0% vs. 42.8%) or major surgery (14.0% vs. 12.9%). The proportion of those who underwent gynecological surgery (5.8% vs. 5.1%), gastrointestinal tract surgery (40.2% vs. 23.9%), urological surgery (9.4% vs. 6.8%), thoracic surgery (5.0% vs. 3.8%) and ophthalmic surgery (1.1% vs. 0.5%) was higher.

### Propensity score matching and cohort balance

PSMs were conducted at a ratio of 1: 1, 1: 2 and 1: 3. After the three PSMs, the distribution of confounders between the CIVA and TIVA groups was relatively balanced, with the SMD distributions depicted in Supplementary Figure S1. Following 1:3 PSM, 1,536 patients were allocated to the CIVA group, and 4,519 patients were allocated to the TIVA group. All matching variables were well balanced, with SMD less than 0.1. The CIVA group exhibited a higher incidence of 3-year (16.1% versus 13.1%) and 3-month (1.8% versus 1.1%) mortality rates compared to the TIVA group (Table 2).

**Table 2:** Data of cancer patients undergoing tumor resection after 1: 3 propensity score matching.

	After propensity score matching				
	TIVA (*N* = 4,519)	CIVA (*N* = 1,536)	*p*-value	SMD	SMD-0.1
Outcomes					
Death 3 years after operation	593 (13.1%)	248 (16.1%)	0.004	–	–
Death 3 months after operation	50 (1.1%)	28 (1.8%)	0.043	–	–
Matching variables					
Age	50.5 (15.5)	50.8 (20.5)	0.644	0.00	<0.1
Male	2,355 (52.1%)	807 (52.5%)	0.796	0.00	<0.1
BMI	23.7 (4.46)	23.6 (5.24)		0.01	<0.1
ASAPS					
I	373 (8.3%)	129 (8.4%)	0.339	0.00	<0.1
II	3,059 (67.7%)	1,011 (65.8%)		0.03	<0.1
III	1,028 (22.7%)	367 (23.9%)		0.02	<0.1
IV and higher	59 (1.3%)	29 (1.9%)		0.05	<0.1
Duration of surgery	3.12 [0, 12.7]	3.22 [0, 13.1]	0.089	0.03	<0.1
Tumor type					
ENT tumor	75 (1.7%)	19 (1.2%)	0.733	0.04	<0.1
Gynecological tumor	307 (6.8%)	91 (5.9%)		0.03	<0.1
Gastrointestinal tract tumor	1764 (39.0%)	610 (39.7%)		0.00	<0.1
Orthopedic tumor	254 (5.6%)	86 (5.6%)		0.00	<0.1
Urological tumor	438 (9.7%)	145 (9.4%)		0.01	<0.1
General surgery tumor	959 (21.2%)	351 (22.9%)		0.05	<0.1
Nervous system tumor	452 (10.0%)	140 (9.1%)		0.03	<0.1
Thoracic tumor	222 (4.9%)	77 (5.0%)		0.00	<0.1
Ophthalmic tumor	48 (1.1%)	17 (1.1%)		0.01	<0.1
Previous surgical history	1,449 (32.1%)	500 (32.6%)	0.748	0.01	<0.1
Previous heart disease	101 (2.2%)	53 (3.5%)	**0.012**	0.04	<0.1
Previous diabetes	284 (6.3%)	109 (7.1%)	0.291	0.02	<0.1
Previous hypertension	965 (21.4%)	337 (21.9%)	0.655	0.00	<0.1
Previous cerebral infarction	80 (1.8%)	26 (1.7%)	0.93	0.01	<0.1
History of drinking	560 (12.4%)	198 (12.9%)	0.642	0.01	<0.1
History of smoking	866 (19.2%)	311 (20.2%)	0.373	0.02	<0.1
Surgery grade					
Intermediate	2,763 (61.1%)	932 (60.7%)	0.532	0.02	<0.1
Major	674 (14.9%)	217 (14.1%)		0.02	<0.1
Minor	1,082 (23.9%)	387 (25.2%)		0.04	<0.1
Emergency surgery	536 (11.9%)	186 (12.1%)	0.831	0.01	<0.1
Shift change of anesthesiologist	891 (19.7%)	329 (21.4%)	0.933	0.01	<0.1
Night shift (18:00 ~ 8:00)	296 (6.6%)	99 (6.4%)	0.161	0.03	<0.1
Transfusion	658 (14.6%)	264 (17.2%)	**0.015**	0.05	<0.1
Intraoperative blood loss	122 (345)	139 (402)	0.142	0.03	<0.1
Intraoperative blood transfusion amount	61.0 (215)	71.6 (288)	0.188	0.03	<0.1
Intraoperative vasoactive drugs	1,112 (24.6%)	375 (24.4%)	0.906	0.02	<0.1

TIVA = total intravenous anesthesia; CIVA = combined inhalation and intravenous anesthesia; SMD=Standard mean deviation; BMI=Body Mass index; ASAPS = American society of Anesthesiologists physical status; ENT surgery = ear, nose, and throat surgery. Data are presented as the number (percentage) for categorical variables and as the mean ± SD or median (minimum, maximum) for continuous variables, as appropriate. The independent samples *t*-test was used to compare continuous variables and the chi-square test to compare categorical variables between groups. *p*-value <0.05 was marked in bold.

### Survival outcomes and association analyses

**Figure 2:**
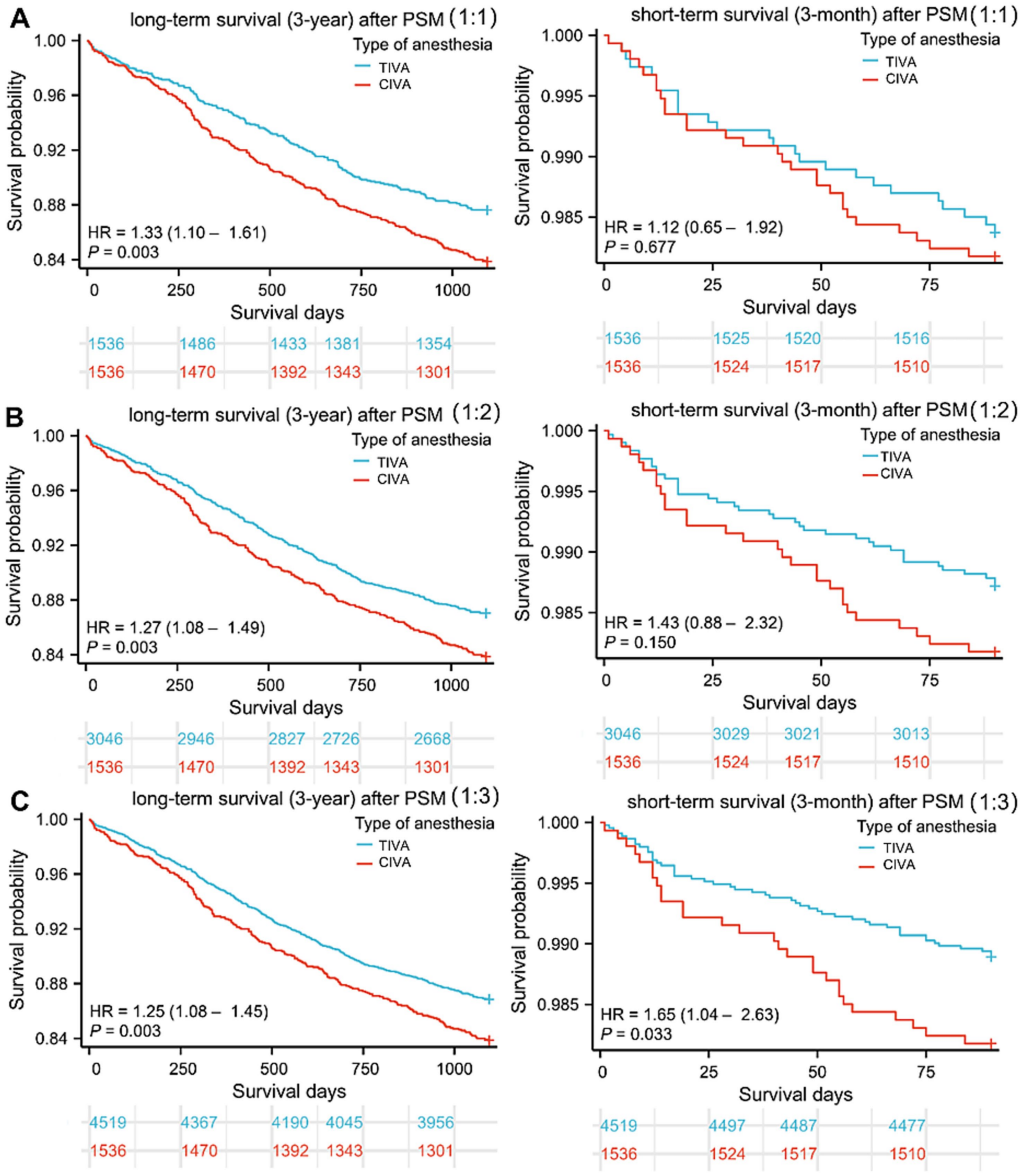
Survival curves after tumor resection. **(A–C)** Three-year and three-month follow-up survival days of patients in CIVA and TIVA group following 1:1, 1:2, and 1:3 PSM. HR: Hazard ratios; CIVA: combined anesthesia; TIVA: total intravenous anesthesia.

As shown in the Figure 2, the survival curve of CIVA group patients was significantly higher than that of TIVA group patients in three PSM cohorts, irrespective of 3-year or 3-month death after cancer surgery. CIVA was significantly associated with reduced 3-year survival, as indicated by statistically significant HR values across all three PSM cohorts. However, CIVA did not exhibit a significant association with the 3-month survival period. The HRs from the various Cox regression models are presented in Table 3. For 3-year postoperative survival, the HR for CIVA remained relatively stable across both the pre-PSM and the three post-PSM cohorts. Prior to PSM, CIVA was associated with a HR of 1.845 (95% CI: 1.620 to 2.101) for 3-year mortality. After PSM, the HR slightly decreased but retained its statistical significance. Following 1:3 matching, there were still differences in cardiac history and blood transfusion between the CIVA and TIVA groups (*p* < 0.05). Consequently, a multivariate Cox regression model was employed for adjustment, and even after adjustment, CIVA was still associated with reduced 3-year survival [HR: 1.210 (95% CI: 1.043–1.404)] but not with 3-month survival [HR: 1.574 (95% CI: 0.990–2.501)].

**Table 3:** Cox regression analysis for long-term survival (three-year) and short-term survival (three-month) in the cancer patients underwent tumor resection.

	Long-term survival (three-year)			Short-term survival (three-month)		
	Hazard Ratio	95% CI	*P*-value	Hazard Ratio	95% CI	*P*-value
Model 1 (before PSM)						
TIVA	Reference			Reference		
CIVA	1.845	1.620, 2.101	**<0.001**	2.318	1.568, 3.425	**<0.001**
Model 2 (After 1: 1 PSM)						
TIVA	Reference			Reference		
CIVA	1.334	1.104, 1.611	**0.003**	1.122	0.654, 1.923	0.677
Model 3 (After 1: 2 PSM)						
TIVA	Reference			Reference		
CIVA	1.269	1.083, 1.487	**0.003**	1.428	0.879, 2.321	0.150
Model 4 (After 1: 3 PSM)						
TIVA	Reference			Reference		
CIVA	1.254	1.081, 1.454	**0.003**	1.654	1.041, 2.627	**0.033**
Model 5 (After 1: 3 PSM and adjusted)						
TIVA	Reference			Reference		
CIVA	1.210	1.043, 1.404	**0.012**	1.574	0.990, 2.501	0.055

TIVA = total intravenous anesthesia; CIVA = combined inhalation and intravenous anesthesia; variables with *P* < 0.05 are included in the multivariate COX regression analysis (Model 4) for adjustment. Bold values indicate statistical significance (*p* < 0.05).

### Subgroup analysis within the CIVA group

To explore heterogeneity, a *post hoc* analysis was performed comparing outcomes between patients receiving CIVA with desflurane (*n* = 489) versus sevoflurane (*n* = 1,072). Baseline characteristics were largely comparable between the two subgroups (Supplementary Table S1), except for a difference in gender distribution (*p* < 0.05). After adjusting for gender in Cox regression models, no statistically significant difference in 3-year survival (adjusted HR: 0.84, 95% CI: 0.65 to 1.09, *p* = 0.19) or 3-month survival (adjusted HR: 0.72, 95% CI: 0.34 to 1.54, *p* = 0.40) was observed between the desflurane and sevoflurane subgroups.

## Discussion

Our retrospective cohort study based on PSM, demonstrated that CIVA may decreased postoperative survival of cancer patients, both in univariate and multivariate Cox regression analyses. Remarkably, although CIVA was found to significantly impact long-term outcomes, its influence on short-term mortality was not conspicuous.

Experimental studies indicate that volatile anesthetics influence tumor biology through multiple pathways. Isoflurane upregulates HIF-1α/2α in renal and prostate cancer cells, enhancing migration and cytoskeletal reorganization—effects attenuated by HIF-1α siRNA or propofol (20, 21). Sevoflurane promotes glioma stem cell proliferation via the HIF pathway (9). In ovarian cancer, sevoflurane and desflurane suppress miR-138 and miR-210, activating HIF-1α and driving proliferation and migration, reversible with miRNA mimics (22). Huitink et al. (23) compared how various volatile anesthetics affect gene expression in brain and breast tumor cells. They found that anesthetics modulate tumor cell genes in a time-dependent and agent-specific manner, likely due to structural or concentration differences. Altered genes included those linked to DNA repair, metastasis, and coagulation (e.g., RBBP8, CENPE, and TFPI). These expression signatures could serve as genetic fingerprints to guide therapy and predict recurrence.

However, propofol can influence the phenotype of tumor cells, inhibiting their proliferation, migration, and invasion. In non-small-cell lung cancer, propofol might inhibit tumor cell proliferation and induce apoptosis via miR-21/PTEN/AKT signaling axis (10). Propofol also inhibited osteosarcoma cell proliferation and invasion *in vitro*, involving TGFβ-1 downregulation (15), and AMPK/FOXO1-mediated cellular autophagy and endoplasmic reticulum stress (24, 25). Anesthetics may further influence tumors indirectly via immune modulation: regulatory T cells can promote recurrence (26, 27). However, a recent study in colorectal cancer patients found no significant difference in circulating immune cell subsets between propofol- and sevoflurane-based anesthesia (28). Some studies also suggest that propofol may promote tumor metastasis, primarily through the following mechanisms: on one hand, it can directly act on key RNA molecules and related signaling pathways, thereby accelerating tumor progression; on the other hand, it may also indirectly foster a tumor-favorable microenvironment by modulating host immune function and influencing the degree of immunosuppression (29).

Although the relationship between anesthetic agents and tumors has been explored extensively over the past two decades, these results, as presented above, are quite diverse, reflecting the complexity of the issue. Numerous clinical studies have also been conducted on this topic. A retrospective research from the United Kingdom discovered that inhalational anesthesia was related with a higher postoperative mortality after multivariable analysis of known variables (7). The 1-year postoperative mortality with volatile anesthesia was roughly 50% higher than with TIVA. However, the study did not take into account the type and stage of cancer, which had a significant impact on prognosis. A study from Taiwan, China, revealed that colon cancer surgery under propofol anesthesia is related with higher survival regardless of tumor-node-metastasis stage (30). This finding was further corroborated by another investigation focusing on hepatocellular carcinoma (14). However, several retrospective studies on Korean populations had come to inconsistent conclusions, finding no significant association between the type of general anesthetic agents used during surgery and long-term survival after cancer surgery (12, 13). A recent study in Sweden investigated the impact of propofol vs. inhalational volatile anesthetics on survival outcomes in stage 1–3 colorectal cancer patients undergoing surgery from 2014 to 2019. The results showed no significant association between the type of anesthetic agent used and long-term survival or disease-free survival in patients with either colon or rectal cancer (31). Nevertheless, all of these investigations were retrospective, which reduced the credibility of the results. Few randomized controlled trials (RCTs) were available for reference. The only well-sized RCT in this area (so far), the CAN-study, reported the results of at least 5 years of follow-up for breast cancer surgery. They found no difference in survival between patients anesthetized with propofol or sevoflurane for breast cancer surgery (32).

Further high-quality RCTs are required to investigate the impact of various general anesthesia techniques on patient survival outcomes. The profound initial imbalance between exposure groups underscores a dominant institutional preference for TIVA at our center during the study period. While PSM successfully balanced measured confounders, the matched cohort represents a selected subset of TIVA patients comparable to the smaller CIVA group. This may limit the generalizability of our findings to centers with different practice patterns, and results should be interpreted as most applicable to patients with clinical profiles similar to those in our CIVA cohort. However, few clinical studies have investigated the postoperative mortality of cancer patients under combined anesthesia regimens. Since this combined approach is common in Chinese practice, we focused on comparing TIVA with CIVA. Our analysis—using PSM-adjusted Cox models—shows that CIVA is associated with an increased risk of three-year mortality after cancer surgery, suggesting that the inclusion of inhalational agents may adversely affect long-term survival. Furthermore, this study focused on general anesthetic techniques and did not evaluate the role of regional anesthesia, which may offer benefits in postoperative recovery and potentially influence oncological outcomes. Future studies should aim to integrate the effects of regional anesthesia techniques to inform a more comprehensive perioperative strategy.

It is worth noting that our study found that CIVA had no significant impact on short-term survival. The reasons for this differential effect on short-term and long-term survival are not entirely clear. One possibility is that the impact of CIVA on long-term survival is mediated through its effects on tumor recurrence, metastasis, or the immune response (20, 22). Inhaled anesthetics have been shown to modulate immune function and may affect the body’s ability to combat cancer progression over time (20, 22). Further research is necessary to elucidate the underlying mechanisms that account for the observed differences in survival outcomes. Prospective studies with detailed follow-up and biological marker analysis could help to clarify the specific pathways through which CIVA impacts long-term survival. Additionally, understanding the potential biological mechanisms could inform the development of targeted interventions to mitigate the adverse effects of CIVA on long-term survival after cancer surgery.

The research does have certain limitations. Firstly, although propensity score matching was employed to address known confounders, this design remains susceptible to biases inherent to non-randomized data. Additionally, the tumor-node-metastasis (TNM) stage, a critical indicator of cancer severity, was not integrated into the multifactorial model. However, due to the fact that TNM was not included as a routine extractable variable in the perioperative database, TNM data was missing in this study. Future studies should consider including TNM variable to enhance the robustness of the analysis. Additionally, detailed data on perioperative pain management strategies, including specific analgesic agents and techniques, were not available in our dataset. Differences in postoperative analgesia could represent a potential unmeasured confounder affecting long-term outcomes. Lastly, within the CIVA group, we lacked data on the precise concentration of the volatile anesthetic used (sevoflurane or desflurane) and the proportional contribution of inhalation versus intravenous agents to the total maintenance dose. This absence precludes an analysis of a dose–response relationship and limits the granularity with which the ‘CIVA’ exposure can be interpreted, as the biological impact may vary with the relative intensity of each component.

## Conclusion

This retrospective analysis suggests an association between anesthetic technique and mortality in patients undergoing cancer surgery. Our results provide indirect evidence of a potential hazard of inhaled anesthetics on long-term survival after cancer surgery, even as a compound in CIVA. However, due to the limitations of this retrospective study, including the lack of tumor TNM staging and other key variables, these findings should be interpreted with caution. Further prospective studies with detailed follow-up and biological marker analysis are needed to elucidate the specific pathways through which CIVA impacts long-term survival.

## Data Availability

The raw data supporting the conclusions of this article will be made available by the authors, without undue reservation.
