# Supplementary material for: Comparison of total intravenous anesthesia and combined inhalation and intravenous anesthesia on survival after tumor surgery: a propensity score matched cohort study
Source: Front Med (Lausanne). 2026 Feb 11;13:1659919. doi: 10.3389/fmed.2026.1659919 (PMC12932620; doi:10.3389/fmed.2026.1659919)
Supplement: Supplementary file 1 [file Data_Sheet_1.docx]

**Supplementary Online Content**

**Comparison of Total Intravenous Anesthesia and Combined Inhalation and Intravenous Anesthesia on Survival After Tumor Surgery: A Propensity Score Matched Cohort Study**

**Yu Wang et al.**

[eMethod 1: Detailed confounding variables, and outcome definitions 2](#_Toc218379169)

[eMethod 2: Detailed statistical analyses 4](#_Toc218379170)

[Figure S1. the Standardized mean differences (SMDs) distribution plot of confounders between the groups after 1:1, 1:2, 1:3 Propensity score matching (PSM) 6](#_Toc218379171)

[Table S1. Baseline characteristics between the desflurane and sevoflurane subgroups within the CIVA group 7](#_Toc218379172)

# eMethod 1: Detailed confounding variables, and outcome definitions

The ethics Committee of Union Hospital, Tongji Medical College, Huazhong University of Science and Technology approved this study. Written informed consent was waived by the same committee, as only aggregated non-identifiable data for patients' personal information were used. We extracted information from electronic clinical medical records of Wuhan Union Hospital.

**1. Detailed matching variables**

1) Sex: Male; Female

2) Age: age at the year of surgery; Unit: years

3) BMI: Body Mass Index: Weight (kg) divided by height (m) squared, Unit: kg/m^2^

4) ASAPS: American society of Anesthesiologists physical status: I; II; III; IV and higher

5) Duration of surgery: Unit: hours

6) Tumor type: containing ear, nose & throat [ENT] tumor; Gastrointestinal tract tumor; Orthopedic tumor; Urological tumor; General surgery tumor; Nervous system tumor; Thoracic tumor; Ophthalmic tumor; Ophthalmic tumor

7) Previous surgical history: yes; no

8) Previous heart disease: yes; no

9) Previous diabetes: yes; no

10) Previous hypertension: yes; no

11) Previous cerebral infarction: yes; no

12) History of drinking: yes; no

13) History of smoking: yes; no

14) Surgery grade: intermediate; major; minor

15) Emergency surgery: yes; no

16) Shift change of anesthesiologist: day shift to night shift or night shift to day shift

17) Night shift: from 18:00~8:00

18) Transfusion: yes; no

19) Intraoperative blood loss: Unit: ml

20) Intraoperative blood transfusion amount: Unit: ml

21) Intraoperative vasoactive drugs: yes; no

**2. Detailed outcome definitions**

Our primary outcome was postoperative cancer-cause mortality. Cancer-cause mortality was defined as death with cancer as the root cause of death, recorded in the Life Registration and Cause of Death Monitoring Room, China CDC, which exclude death directly caused by cardiogenic disease and trauma. According to the survival time of patients, the death outcome is further divided into short-term death (3 months after operation) and long-term death (3 years after operation).

# eMethod 2: Detailed statistical analyses

**1. Sample size calculation**

The sample size was based on the available data from all patients accepted cancer surgery at our institution from January 2014 to December 2018. No statistical power calculation was performed before the study.

**2. Propensity score matching**

Given that there were potential confounding variables in this study, propensity score matching was used to construct a weighted cohort of patients which were similar in demographics and surgery characteristics among TIVA and CIVA groups. Matching was performed using 1: 1, 1: 2, and 1: 3 matching protocol without replacement, with a caliper of 0.05 SD of the logit of the propensity score. The propensity score matching was conducted in the R environment, version R version 4·2·0 (April, 2022), with “MatchIt” package.

The clinical data were preliminarily tested for differences using Student’s/Welch’s t-test, or Wilcoxon rank sum exact test for continuous data; Pearson's Chi-squared test, or Fisher's exact test for categorical variables among TIVA and CIVA groups before and after propensity score matching.

The clinical data are presented as the number (percentage) for categorical variables and as the mean ± SD or median (minimum, maximum) for continuous variables, as appropriate.

**3. Univariate and multivariable COX regressions**

Univariate COX regression models were used to explore the variables associated with postoperative death and calculated the hazard ratios (HR) together with 95% confidence interval (CI). Then the clinical variables with P < 0.05 entered into multivariable COX regression to calculated the adjusted HR.

# Figure S1. the Standardized mean differences (SMDs) distribution plot of confounders between the groups after 1:1, 1:2, 1:3 Propensity score matching (PSM)

BMI=Body Mass index; ASAPS=American society of Anesthesiologists physical status; ENT surgery=ear, nose, and throat surgery.


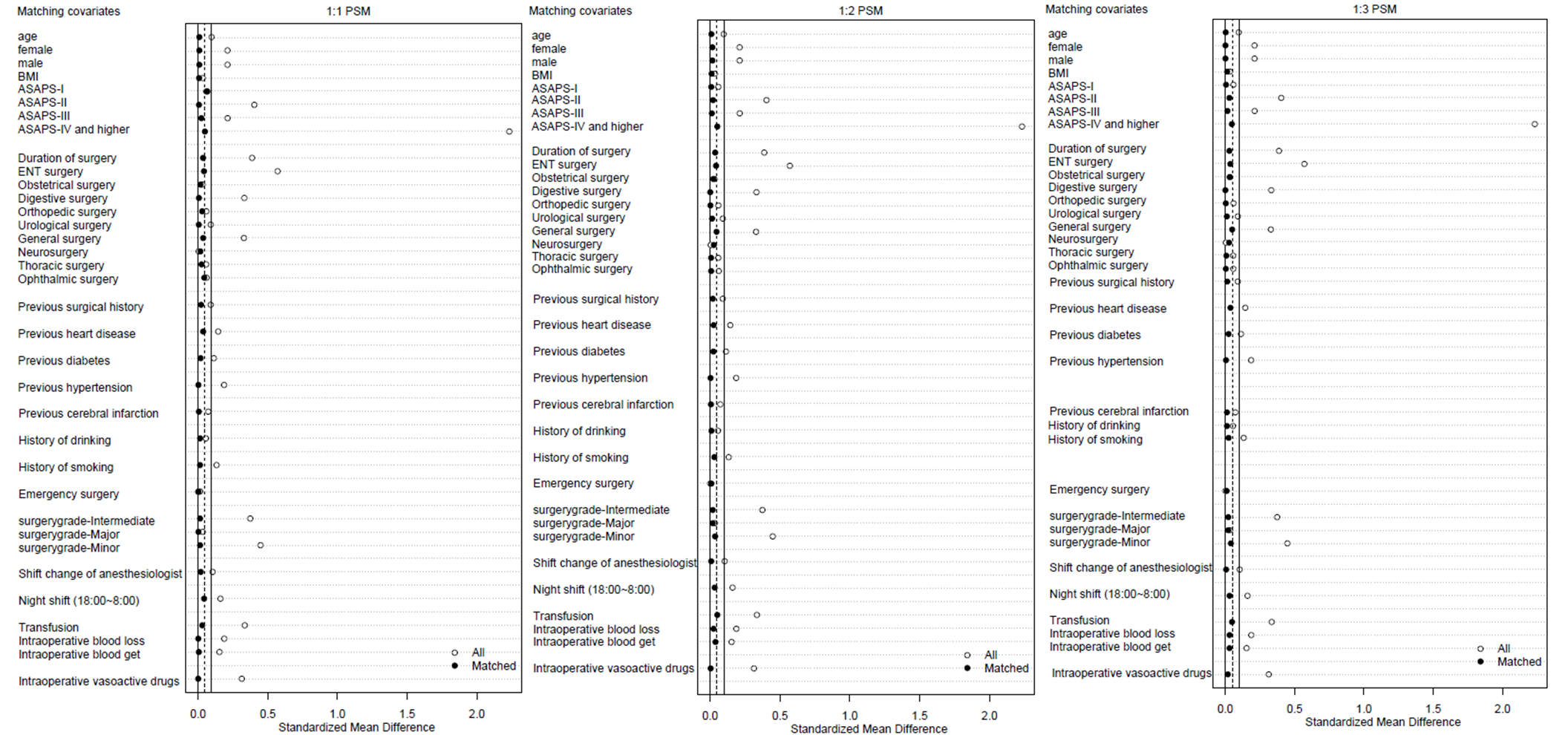


# Table S1. Baseline characteristics between the desflurane and sevoflurane subgroups within the CIVA group

|  | Desflurane (N=489) | Sevoflurane (N=1,072) | P-value |
| --- | --- | --- | --- |
| **Outcomes** |  |  |  |
| Death 3 years after operation | 87 (17.8%) | 167 (15.6%) | 0.305 |
| Death 3 months after operation | 11 (2.2%) | 18 (1.7%) | 0.567 |
| Baseline **variables** |  |  |  |
| Age | 50.9 (21.0) | 51.1 (20.2) | 0.881 |
| Male | 235 (48.1%) | 589 (54.9%) | 0.013 |
| BMI | 23.7 (5.26) | 23.6 (5.20) | 0.780 |
| ASAPS |  |  |  |
| I | 40 (8.2%) | 89 (8.3%) | 0.591 |
| II | 315 (64.4%) | 707 (66.0%) |  |
| III | 124 (25.4%) | 251 (23.4%) |  |
| IV and higher | 10 (2.0%) | 25 (2.3%) |  |
| Duration of surgery | 3.27 [0, 13.1] | 3.23 [0.17, 13.1] | 0.566 |
| Tumor type |  |  |  |
| ENT tumor | 5 (1.0%) | 14 (1.3%) | 0.427 |
| Gynecological tumor | 34 (7.0%) | 57 (5.3%) |  |
| Gastrointestinal tract tumor | 196 (40.1%) | 431 (40.2%) |  |
| Orthopedic tumor | 25 (5.1%) | 62 (5.8%) |  |
| Urological tumor | 38 (7.8%) | 109 (10.2%) |  |
| General surgery tumor | 110 (22.5%) | 244 (22.8%) |  |
| Nervous system tumor | 47 (9.6%) | 94 (8.8%) |  |
| Thoracic tumor | 25 (5.1%) | 53 (4.9%) |  |
| Ophthalmic tumor | 9 (1.8%) | 8 (0.7%) |  |
| Previous surgical history | 162 (33.1%) | 347 (32.4%) | 0.811 |
| Previous heart disease | 20 (4.1%) | 46 (4.3%) | 0.962 |
| Previous diabetes | 35 (7.2%) | 79 (7.4%) | 0.965 |
| Previous hypertension | 117 (23.9%) | 236 (22.0%) | 0.440 |
| Previous cerebral infarction | 9 (1.8%) | 19 (1.8%) | 1 |
| History of drinking | 64 (13.1%) | 137 (12.8%) | 0.931 |
| History of smoking | 95 (19.4%) | 223 (20.8%) | 0.577 |
| Surgery grade |  |  |  |
| Intermediate | 293 (59.9%) | 659 (61.5%) | 0.811 |
| Major | 72 (14.7%) | 147 (13.7%) |  |
| Minor | 124 (25.4%) | 266 (24.8%) |  |
| Emergency surgery | 57 (11.7%) | 131 (12.2%) | 0.815 |
| Shift change of anesthesiologist | 109 (22.3%) | 232 (21.6%) | 0.825 |
| Night shift (18:00~8:00) | 32 (6.5%) | 68 (6.3%) | 0.969 |
| Transfusion | 86 (17.6%) | 195 (18.2%) | 0.828 |
| Intraoperative blood loss | 178 (552) | 138 (376) | 0.151 |
| Intraoperative blood transfusion amount | 77.9 (322) | 74.1 (279) | 0.824 |
| Intraoperative vasoactive drugs | 138 (28.2%) | 257 (24.0%) | 0.084 |

Note: BMI=Body Mass index; ASAPS=American society of Anesthesiologists physical status; ENT surgery=ear, nose, and throat surgery. Data are presented as the number (percentage) for categorical variables and as the mean ± SD or median (minimum, maximum) for continuous variables, as appropriate. The independent samples t test was used to compare continuous variables and the chi-square test to compare categorical variables between groups.
